# Influence of Different Types of Fillers on the Performance of PMMA-Based Low-Temperature Rapid Repair Mortar

**DOI:** 10.3390/ma17122871

**Published:** 2024-06-12

**Authors:** Zhipeng Zhu, Lingling Xu, Min Deng, Shijian Lu, Zemeng Guo, Luchao Yan, Yang Wang

**Affiliations:** College of Materials Science and Engineering, Nanjing Tech University, Nanjing 211816, China; 202161103006@njtech.edu.cn (Z.Z.); 202161103065@njtech.edu.cn (S.L.); 202061103028@njtech.edu.cn (Z.G.); 202161203204@njtech.edu.cn (L.Y.); 202261103012@njtech.edu.cn (Y.W.)

**Keywords:** PMMA repair mortar, filler, working performance, mechanical performance, durability performance

## Abstract

In order to further optimize the performance of PMMA (Polymethyl Methacrylate) repair mortar. In this paper, fly ash, talcum powder and wollastonite powder are used as fillers to modify the PMMA repair mortar. The effects of these three fillers on the working performance, mechanical performance and durability of PMMA repair mortar were explored. The study shows that the three fillers have good effect on the bond strength of the repair mortar, in which the fly ash has the best effect on the mechanical performance. The mechanical properties of PMMA repair mortar were best when the amount of fly ash was 60 phr (parts per hundred, representing the amount of the material added per hundred parts of PMMA). At this time, the 28 d compressive strength was 71.26 MPa and the 28 d flexural strength was 28.09 MPa, which increased by 13.31% and 15.33%, respectively. Wollastonite powder had the least negative effect on the setting time of the PMMA repair mortar. When the dosage of wollastonite powder was increased to 100 phr, the setting time was only extended from 65 min to 94 min. When the talc dosage was 60 phr, the best improvement in salt freezing resistance was achieved. After 100 cycles of salt freezing, the mass loss rate and strength loss rate decreased to 0.159% and 4.97%, respectively, which were 75.1% and 37.7% higher than that of the control group. The addition of all three fillers reduced the porosity and the proportion of harmful pores in the mortar. This study contributes to a comprehensive understanding how different types of fillers affect PMMA repair mortars, and it also provides theoretical support for the further development of low-temperature rapid repair mortars.

## 1. Introduction

With the increase in vehicle driving speeds, heavy transportation and the impact of extreme weather, the number of concrete pavement damages and the degree of damage are increasing year by year, which leads to an increasingly severe situation of road maintenance [1]. The common damage of cement concrete pavement can be divided into structural damage and non-structural damage. The latter is often referred to as thin-layer disease, and its main forms of damage are categorized into cracks (cracks on the road surface), exposed bones (exposure of aggregates inside the concrete), pitted surfaces (the loss of bonding material on pavement surface) and surface potholes (deteriorated road surfaces with potholes) [2,3,4]. The method used for repairing thin-layer damage is to pave a new thin layer of repair material in the damaged area, so that the old and new pavements are bonded to each other to form a unified whole. Ultimately, the effects of repeated loads and changes in the external environment on the pavement are shared [5].

The most direct method of repair is to place a layer of cement mortar or concrete with the same material as the old concrete pavement over the damaged area. However, this method has a long maintenance time. Ordinary silicate cement mortar is prone to brittle fracture and high shrinkage, which is not conducive to the compatibility between the repair mortar and the concrete pavement. In order to solve this problem, some scholars added various types of fibers or used early-strength or fast-hardening special cement for modification and optimization [6,7,8,9,10,11,12,13], which greatly improved the toughness of the repair mortar and shortened the maintenance time. Another part of scholars choosed to use epoxy resin, polyurethane, methyl methacrylate and other polymers as repair materials to replace cement mortar [14,15,16,17]. Compared with inorganic repair materials, polymer repair materials have higher bond strength and durability, and can be cured quickly, but its cost is also several times higher than inorganic repair materials [15]. Therefore, some scholars proposed the method of composite organic repair materials with inorganic repair materials. The composite repair material they developed fully combines the advantages of organic and inorganic materials. This composite repair material not only significantly improves the mechanical performance and durability of the repair material, but also reduces the cost significantly compared to pure organic repair materials. It is currently a popular material used to repair surface defects in concrete structures [18,19,20].

Composite repair materials are subdivided into three categories: polymer impregnated mortar/concrete (PIM/PIC), polymer-modified mortar/concrete (PMM/PMC) and polymer mortar/concrete (PM/PC). PM/PC consists only of aggregates and polymers, and it uses polymers as the binder, which drastically reduces the curing time compared to ordinary cement concrete. PM/PC not only has excellent strength and durability, but also has good bonding ability with the substrate, so it is widely used in pavement repair projects. The commonly used polymers in PM/PC are epoxy resin, polyurethane and unsaturated polyester, etc., and the repair materials made from them have good mechanical performance and durability [21,22,23,24]. Methyl methacrylate (MMA) has the advantages of good fluidity and strong adhesion. Moreover, it has a wider temperature range [25,26,27] that can be used in temperatures ranging from −30 °C to 45 °C. Using MMA as the binder material for the repair mortar is expected to provide a composite repair mortar that cures quickly at low temperatures and has high strength for thin-layer lesions on pavements in winter or cold regions.

However, it was found during the study that PM/PC undergoes an exothermic reaction of monomer polymerization during the setting process. The high temperatures resulting from the exothermic reaction cause a large difference in the coefficient of thermal expansion between PM/PC and the concrete substrate. The bonding interface between PM/PC and the substrate can also be subject to large stresses at the interface due to temperature changes, resulting in poor thermal compatibility between the repair material and the substrate [28,29]. In order to solve this problem, researchers have found that the addition of fillers can reduce the coefficient of thermal expansion and modulus of elasticity of polymer mortar through extensive experiments [30]. The smaller particle size of the filler can uniformly fill the internal pores of the repair mortar, thus preventing the expansion of micro-cracks and increasing cohesion. Besides, the addition of filler can also reduce the cost of the polymer mortar.

Rebeiz and Gorninski et al. [31,32] investigated the effect of fly ash filler incorporation on unsaturated polyester mortar. The results showed that the incorporation of fly ash not only significantly improved the mechanical properties and bond strength of the polymer mortar, but also reduced the coefficient of thermal expansion. Pham [33] studied the effect of cement on the mechanical strength of polymer concrete when used as a filler. The findings showed that the filler significantly increased the flexural and compressive strength of polymer concrete. They used a tensile testing machine to test the bond strength of a 300 mm thick PC layer to a substrate. It was found that at low polymer dosages, fillers enhanced the bonding strength. However, at higher polymer levels, the bonded specimens fractured on the substrate with or without fillers. This indicates that the bonding is effective. Guanjie Li et al. [34] analyzed the changes in the performance of epoxy repair mortars filled with different forms of fillers. It was found that any form of filler could improve the densification of the epoxy mortar, thus increasing its compressive and tensile bond strengths and reducing its shrinkage. Among them, the spherical particles of coal gasifier slag can transfer the internal stress of the mortar more uniformly, so its improvement effect on the densification of epoxy mortar is the most significant. Chandrasekaran [35] studied the effect of heavy calcium carbonate and fly ash on the performance of unsaturated polyester mortar. It was finding that spherical fly ash enhanced the PM strength more significantly with almost zero water absorption as compared to heavy calcium carbonate. Yemam [36] investigated the effect of sand washing waste on epoxy resin mortar. The results showed that a certain amount of sand washing waste could significantly improve the mechanical properties and bond strength of epoxy mortar. They concluded that sand washing waste can be used as a potential filler for epoxy resin mortar, but specific issues such as dimensional compatibility need to be further explored.

In this study, PMMA repair mortar for quick repair at low temperature of −10 °C is used as the base proportion. Fly ash in micro-spherical form, talcum powder in flake form and wollastonite powder in pin-rod form were used as fillers. By changing the amount of fillers, the effects of each form of fillers on the working performance, mechanical properties and durability of PMMA repair mortar were investigated. The structural changes of PMMA repair mortar after the addition of fillers were also analyzed by microscopic morphology. It is expected to obtain the best choice of filler type and dosage to optimize the performance of PMMA repair mortar and reduce the cost, so as to lay the foundation for its wider application.

## 2. Materials and Experiments

### 2.1. Raw Materials

**Figure 1:**
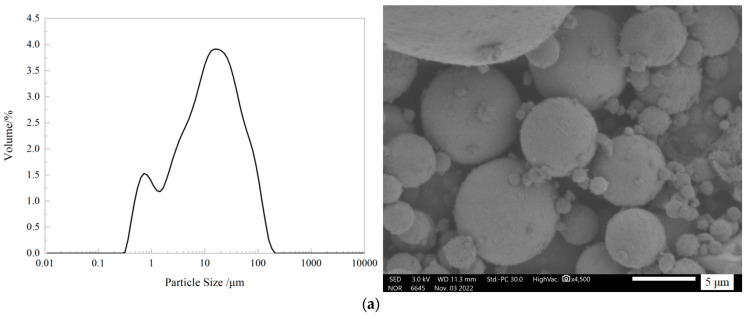

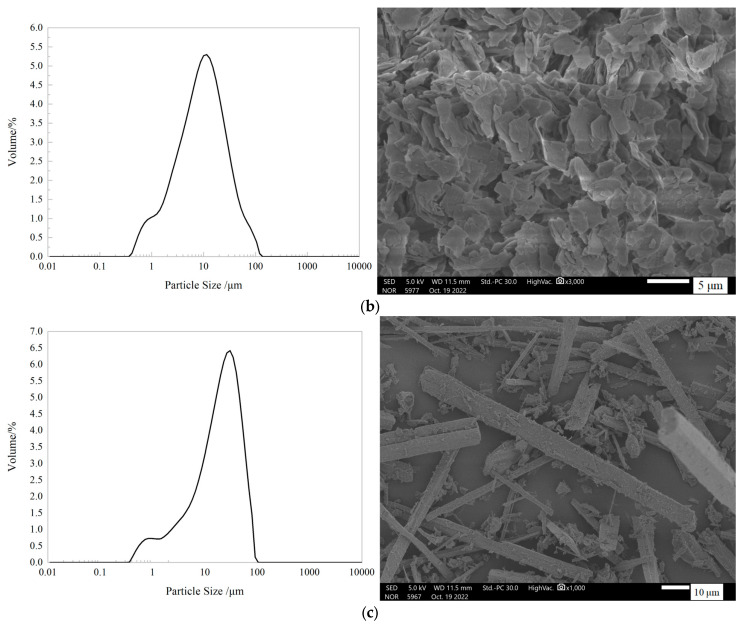
Particle size distribution and microscopic morphology of fillers. (**a**) fly ash (**b**) talc (**c**) wollastonite.

MMA prepolymer was prepared by using methyl methacrylate as polymerization monomer, benzoyl peroxide (BOD) as initiator, dioctyl phthalate (DOP) as the plasticizer, N, N-dimethylaniline (DMA) as curing agent, and polyvinyl acetate (PVAc) + styrene as low shrinkage additives. Bagged ISOA standard sand from Xiamen Aceo Standard Sand Co., Ltd. was used as fine aggregate. Spherical fly ash, lamellar talc and needle-and-rod wollastonite powder were used as fillers, and the particle size distribution map, micro-morphology map and particle size distribution data are shown in Figure 1 and Table 1. The average particle size of fly ash, talcum powder and wollastonite powder are 11.55 μm, 8.82 μm and 18.58 μm. The γ-methacryloxypropyltrimethoxysilane (KH570) from Shanghai Yuanye Biotechnology Co. Ltd. (Shanghai, China) was used as the silane coupling agent.

### 2.2. Specimen Preparation

#### 2.2.1. Preparation of MMA Prepolymers

Through the preliminary study [37], 12% of (PVAc+ Styrene) was selected as the low shrinkage additive. The dosage is the percentage of the mass of the solution formed by PVAc dissolved in styrene to the total mass of the whole MMA prepolymer. The distribution of each component of the prepared MMA prepolymer is shown in Table 2. Finally, the MMA prepolymer is a light yellow transparent and homogeneous fluid, free of impurities, without precipitation and delamination.

#### 2.2.2. Preparation of PMMA Repair Mortar

The basic ratio of PMMA repair mortar is PMMA:DMA:standard sand:silane coupling agent = 100:4:550:0.75 (mass ratio),its performance is shown in Table 3. The difference is the addition of different types of fillers in different dosages. Different dosages of fly ash, talc and wollastonite powder were used as fillers to modify the PMMA repair mortar. The amount of filler is defined on the basis of 100 parts of PMMA, i.e., when the amount of filler is 40 parts, the mass ratio of filler:PMMA is 40:100. Phr is parts per hundred, which stands for the amount of the substance added per hundred parts of PMMA. F1, F2, F3, F4, and F5 represent fly ash in dosages of 20, 40, 60, 80, and 100 parts, and similarly T1–5 and W1–5 represent talc and wollastonite powders in dosages of 20–100 parts.

In order to simulate the real construction process. Prior to the preparation of the PMMA repair mortar, the MMA prepolymer, curing agent and silane coupling agent KH570 prepared in Section 2.2.1 were left at −10 °C for 24 h and taken out before preparing the mortar. When preparing the repair mortar, the MMA prepolymer is first added to the corresponding proportion of hardener and silane coupling agent and stirred well. Then, the sand, filler and mixed solution are poured into the mixing pot and stirred into shape. After molding, put the specimen together with the mold into the curing box at −10 °C, take off the mold after 24 h and put it into the curing box at −10 °C again to curing to the corresponding age.

### 2.3. Testing Method

#### 2.3.1. Mechanical Strength of Repair Mortars

After the resulting PMMA repair mortar was maintained to the corresponding test age, the flexural strength and compressive strength were tested with reference to GB/T 17671-2021 [38]. The compressive strength and flexural strength of the specimens were determined with WDW-20E microcomputer-controlled electronic universal testing machine under the loading speeds of 2.4 kN/s and 0.05 kN/s. The test results were as follows.

#### 2.3.2. Fluidity

Referring to GB/T 2419-2005 [39], NLD-3 cementitious sand fluidity tester (Wuxi Jianyi Building Material Instrument Factory, Wuxi, China) was used to conduct the test. Firstly, the PMMA repair mortar was divided into two layers and quickly put into the test mold and pounded. After pounding, the test mold was gently lifted upward and immediately rotated on the beating table, completing 25 beatings in 25 ± 1 s at a frequency of once per second. After jumping, use a steel ruler to measure the diameter of the bottom of the PMMA repair mortar perpendicular to each other in both directions, and the average of the two diameters obtained is the flow of the PMMA repair mortar.

#### 2.3.3. Setting Time

(1) The setting time of the polymer PMMA was determined by filling the test tube with the polymer solution. Start timing from the MMA prepolymer add curing agent mixing, pour the solution into the test tube, tilt the test tube from time to time. When the liquid no longer flows while the test tube is placed flat, which means that the liquid in the test tube loses its fluidity, the timing is stopped. And this time is the setting time of PMMA.
(2) Determination of setting time of PMMA repair mortar with reference to JGJ/T 70-2009 [40]. Fill and smooth the fresh repair mortar mix into a slurry container with an inner diameter of 140 mm and a height of 75 mm, and place it in a maintenance box at −10 °C for maintenance. It was taken out every 10 min and tested using ZKS-100 mortar setting time tester (Tianjin Beichen Testing Instrument Factory, Tianjin, China). The penetration resistance method was used to determine the setting time of the PMMA repair mortar by the resistance generated by the mortar to the penetrating test needle, which was measured every 2 min as it approached the setting time.

#### 2.3.4. Bonding Strength

**Figure 2:**
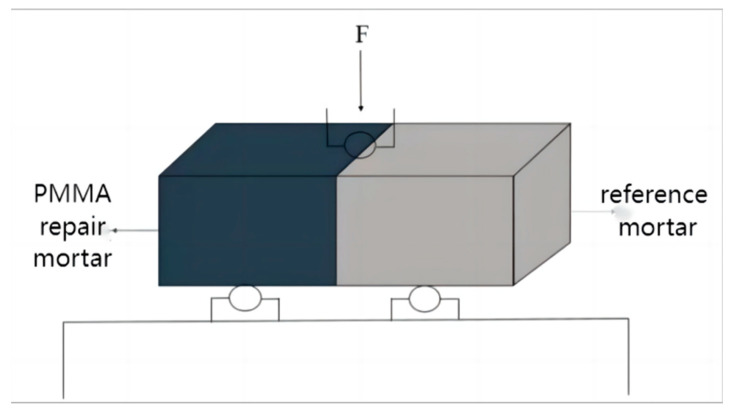
Schematic diagram of flexural bonding experiment.

**Figure 3:**
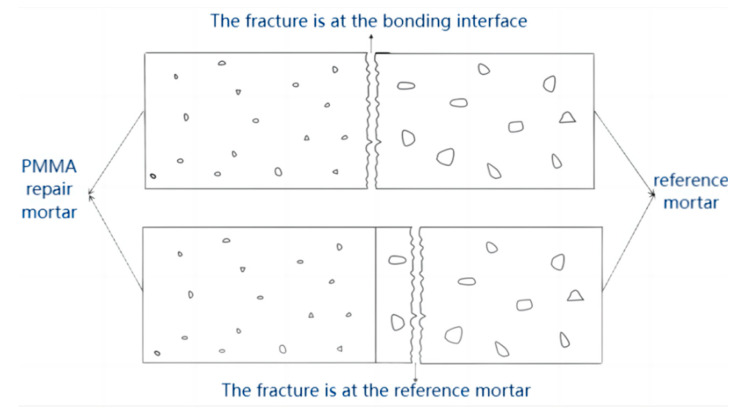
Schematic diagram of the two types of fracture interfaces.

The baseline mortar specimens were broken in the center after being cured for 7 d under standard curing conditions. Put half of the broken specimen into a mold with the size of 40 mm × 40 mm × 160 mm, and pour PMMA repair mortar into the other side of the mold. Finally, the resulting specimens were cured at −10 °C until 28 d. After the maintenance was completed, the flexural bond strength of the bonded specimen was tested according to the test method of flexural strength introduced in Section 2.3.1 as shown in Figure 2. The two fracture surfaces of the specimen after fracture are shown in Figure 3. If the fracture surface occurs at the bond interface between the repair mortar and the reference mortar, the resulting flexural strength is the bond strength of the repair mortar. But if the bond specimen fractures on the reference mortar specimen, the bond strength of the repair mortar is greater than the value of the resulting flexural strength.

#### 2.3.5. Abrasion Resistance

The abrasion resistance test was carried out with reference to GB/T 3810.6-2016 [41], and the abrasion volume and abrasion mass were used to characterize the abrasion resistance of the material. The abrasion mass was determined by the mass difference of the specimen before and after the abrasion resistance test, and the abrasion pit volume was calculated from the measured chord length of the abrasion pit through Equations (1) and (2):

(1) $$V=\left(\frac{\pi \cdot \alpha }{180}-\sin\alpha \right)\times \left(\frac{h\times d^{2}}{8}\right)$$

(2) $$\sin\frac{\alpha }{2}=\frac{L}{d}$$

where the V is the volume of the grinding pit (mm^3^), the L is the chord length of the grinding pit (mm), the α is the angle formed between the chord length of the grinding pit and the center of the friction wheel (°), the h is the thickness of the friction wheel, which is 10 mm in the present test, and d is the diameter of the friction wheel, which is 200 mm in the test.

#### 2.3.6. Water Absorption Capability

Referring to the test method in DL/T5126-2021 [42] for determination. The specimen was cured in the curing box at −10 °C until 28 d, and then put into the oven at 80 ± 2 °C for 48 h and taken out, then put in the desiccator to cool to room temperature and quickly weigh the weight of the specimen at this time, G_0_, and then put the specimen into the water at 20 ± 2 °C for 48 h. During the immersion process, the upper surface of the specimen should be more than 50 mm away from the water surface. After the end of the immersion with a wet cloth to wipe off the surface water and quickly weigh the weight of the test piece G_1_. Finally, the water absorption rate is calculated according to the Equation (3):

(3) $$W=\frac{G_{1}-G_{0}}{G_{0}}\times 100\%$$

where W is the water absorption rate (%) of the specimen; G_0_ is the mass of the specimen after drying (g); G_1_ is the mass of the specimen after water absorption (g).

#### 2.3.7. Salt Scaling Resistance

The PMMA repair mortar specimens were cured in a curing box at −10 °C for up to 24 d. Subsequently, the specimens were immersed in a salt solution configured with a mass ratio of 97% distilled water and 3% NaCl at 20 °C for 4 d. Remove the specimens immediately after the end of immersion, dry the surface with a damp cloth and weigh it. Each salt freezing cycle should be frozen in a freezer at −20 °C for more than 4 h and then immediately thawed in salt solution at 20 °C for more than 4 h. The specimens were weighed and tested for compressive strength at the end of every 25 cycles. The rate of loss of compressive strength and the rate of mass loss of the PMMA repair mortar were used to express its resistance to salt freezing. The rate of loss of compressive strength is calculated in Equation (4):

(4) $$∆f_{c}=\frac{f_{c0}-f_{cn}}{f_{c0}}\times 100$$

where Δf_c_ is the compressive strength loss rate (%) of the repair mortar after N salt freezing cycles, f_c0_ is the compressive strength measurement value (MPa) of the repair mortar in the blank group without salt freezing cycles, and f_cn_ is the compressive strength measurement value (MPa) of the repair mortar after N salt freezing cycles.

The rate of mass loss is calculated according to Equation (5):

(5) $$∆W_{ni}=\frac{W_{0i}-W_{ni}}{W_{0i}}\times 100$$

where ΔW_ni_ is the mass loss rate (%) of the i-th mortar specimen after N salt freezing cycle, W_0i_ is the mass (g) of the i-th mortar mortar before salt freezing cycle, and W_ni_ is the mass (g) of the i-th mortar specimen after N salt freezing cycle.

#### 2.3.8. Volumetric Deformation

Referring to JC/T 603-2004 [43], PMMA repair mortar was filled into 25 mm × 25 mm × 280 mm molds that had been pre-assembled with measuring nail heads. After 24 h of curing in the curing box at −10 °C and demolding, the initial length L_0_ of the mortar should be determined by a specific length meter within 30 min after demolding, and then continue to be placed at −10 °C for curing to different ages to measure its length. The formula for shrinkage at different ages is:

(6) $$S_{n}=\frac{L_{0}-L_{n}}{L_{e}}\times 100$$

where S_n_ is the shrinkage (%) of the specimen at different age n, L_0_ is the initial length of the specimen (mm), L_n_ is the length of the specimen at different age n (mm), and L_e_ is the effective length of the specimen with the nail head portion removed, which is 250 mm in the present experiment.

#### 2.3.9. Pore Size Distribution

The internal pore size distribution of PMMA repair mortar was tested by using mercuric pressure method. The operation steps are shown below:

(1) Use a firm hammer to knock the PMMA repair mortar specimen into 3~5 mm size granular sample;
(2) Put it into a constant temperature blower drying oven to dry the moisture;
(3) Put the sample into the Quantachrome PoreMaster 60 mercury pore analyzer for testing.

#### 2.3.10. Scanning Electron Microscopy

A scanning electron microscope (SEM) model JSM-5900 was used to observe and analyze the internal structure of the PMMA repair mortar and the bonding interface between the PMMA repair mortar and the reference mortar in terms of microscopic morphology. The test procedure is shown below:

(1) Crack the PMMA repair mortar and select the particles of which the diameter is about 10 mm;
(2) Place the selected mortar particles in a constant temperature blast drying oven to dry the moisture;
(3) Use conductive adhesive to fix the sample to be tested in the sample stage, to be coated and processed into the scanning electron microscope observation.

## 3. Results and Discussions

### 3.1. Working Performance

**Figure 4:**
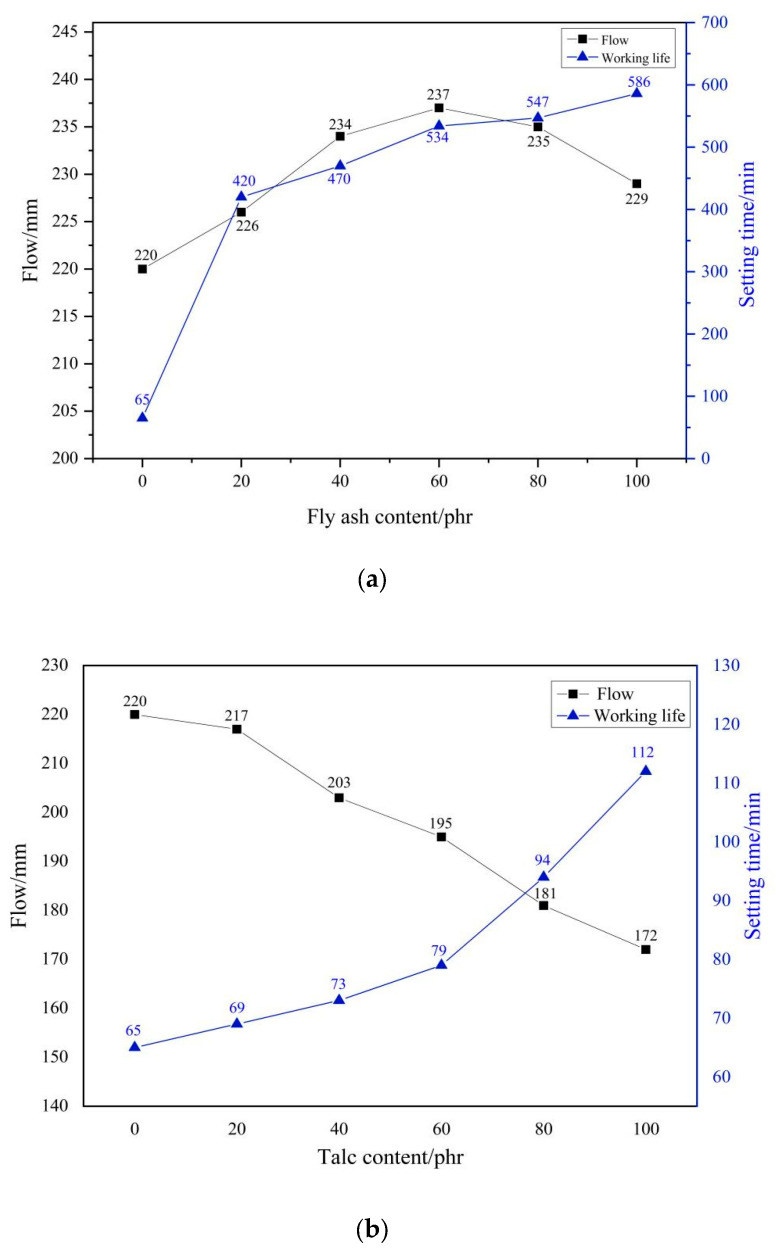

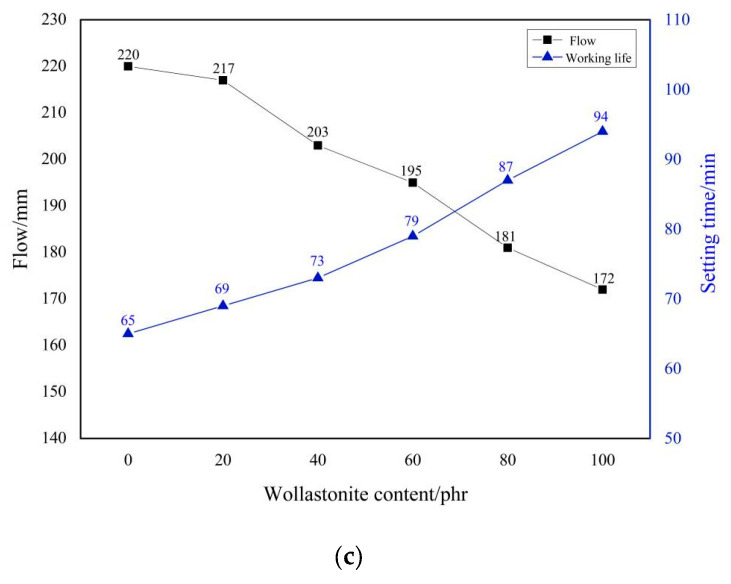
Influence of fillers contents on the workability of PMMA repair mortar. (**a**) fly ash (**b**) talc (**c**) wollastonite.

Figure 4 shows the effect of filler on the working performance of PMMA repair mortar. It can be seen that the increase in the amount of filler significantly affects the working performance of the mortar system. Unlike talc and wollastonite powder, the flowability of the mortar shows a tendency to increase and then decrease as the amount of fly ash increases. his is due to the spherical fly ash has a rolling ball effect, which enhances the wetting ability of PMMA, so with the increase of fly ash dosage, the ease of PMMA repair mortar can be improved. However, when the dosage of fly ash is too much, the fly ash particles will appear localized agglomeration phenomenon, so the fluidity is reduced again. Talc and wollastonite powder, due to the special morphology of lamellar and needle and rod on their surfaces, respectively, make them adsorb more PMMA while filling the internal voids of the PMMA repair mortar, so the proportion of PMMA used in the bonding aggregate decreases, thus reducing the fluidity of the repair mortar.

In addition, all three fillers dosages extended the setting time of the PMMA repair mortar at −10 °C environment. When adding more fillers, the lower the proportion of PMMA components in the mortar system that play a cementing role, the smaller the exothermic effect during the reaction, the lower the speed of the final formation of a continuous polymer network, which leads to prolonging the setting time of the mortar. The setting time of PMMA repair mortar with fly ash, talc and wollastonite powder increased from 65 min to 586 min, 112 min and 94 min, respectively, when the dosage of the three fillers was increased to 100 phr. In addition, at the same dosage, the effect of fly ash in retarding the setting was much stronger than that of the other two fillers. This is also related to the unique spherical particles of fly ash, which have a smooth surface and play a certain dispersing effect on PMMA, making the intermolecular distance of PMMA larger, thus substantially delaying the setting time of PMMA repair mortar. Wollastonite powder has the least effect on the setting time of PMMA repair mortar, which is more suitable to be used in the project of rapid repair of pavement.

### 3.2. Mechanical Performance

#### 3.2.1. Flexural and Compressive Strength

**Figure 5:**
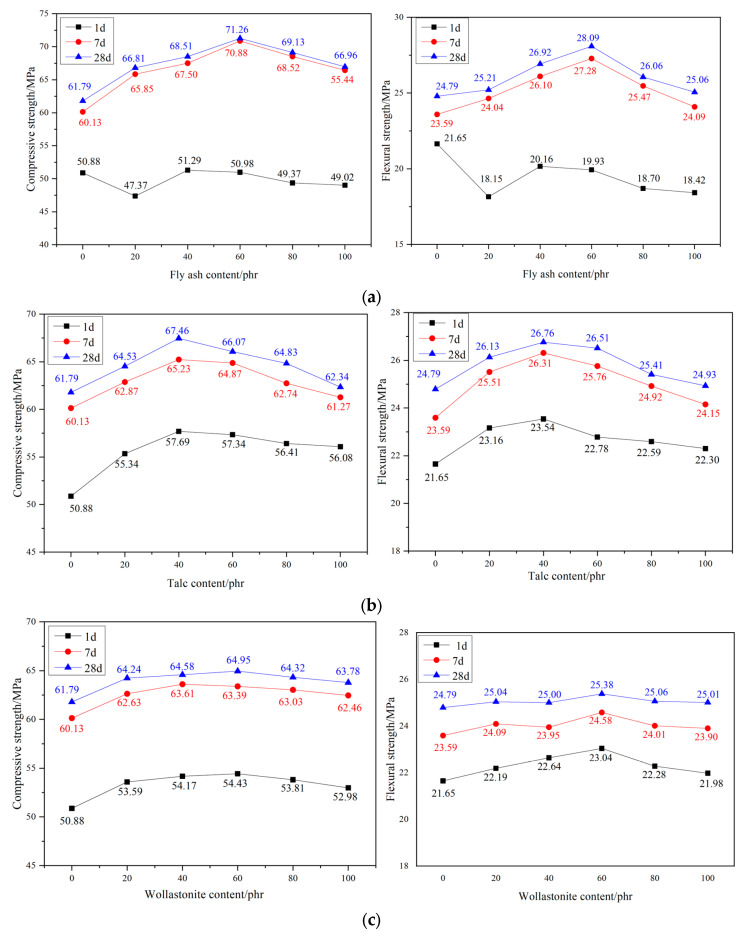
Influence of fillers contents on the mechanical property of PMMA repair mortar. (**a**) fly ash (**b**) talc (**c**) wollastonite.

From Figure 5, it can be seen that the strength of PMMA repair mortar increases and then decreases with the increase of filler dosage. The addition of all three fillers increased the flexural and compressive strength of PMMA repair mortar at 28 d to different degrees. When the dosage of fly ash was 60 phr, it had the best reinforcing effect, and the flexural and compressive strengths at 28 d reached 28.09 MPa and 71.26 MPa, which were 13.31% and 15.33% higher than that of the reference group. When the dosage of talc was 40 parts, there was an increase in flexural and compressive strength at 28 d by 7.94% and 8.32%. At 60 phr of wollastonite powder, the increased flexural and compressive strength of PMMA repair mortar at 28 d compared to the reference group were 2.38% and 5.13%. In summary, Dosing of fly ash at 60 phr had the most significant effect on the improvement of mechanical performance of PMMA repair mortar. Compared with lamellar and needle-and-rod particles, microspherical particles can more evenly transfer the external force to the resin matrix, reducing the stress concentration inside the PMMA repair mortar, thus reducing or delaying the generation and expansion of microcracks.

Since fly ash substantially prolongs the setting time of mortar, its 1 d early strength is reduced when used as a filler compared to no filler. However, its enhancement is best in the later stages of strength development. Talc and wollastonite powders were more effective in enhancing the 1 d early strength than the 28 d late strength. This may be due to the fact that in the early stage of the maintenance of PMMA repair mortar, there is still some MMA that has not reacted with the curing agent, so the filler contributes more to the strength of PMMA repair mortar. At the late stage of maintenance, the PMMA has been cured completely. At this time, although the addition of filler can still increase the strength of PMMA repair mortar, but the enhancement effect is not as obvious as in the early stage.

#### 3.2.2. Bond Strength

In addition to the mechanical performance of PMMA repair mortar itself, the bond strength between the repair mortar and the reference mortar is also an important factor affecting the actual use of PMMA repair mortar. From Table 1, it is known that the base proportion of PMMA repair mortar has good bonding performance with the reference mortar, so it is also necessary to study the effect of the addition of filler on the bonding performance of PMMA repair mortar under the temperatures at −10 °C, the results are shown in Table 4.

As can be seen in Table 4, the addition of filler did not reduce the flexural bond strength between the PMMA repair mortar and the reference mortar. The fracture surfaces of all the filler-added bond specimens were still located on the reference mortar specimen. This indicates that the flexural bond strength of the filled PMMA repair mortar is 5.5 MPa higher than that of the reference mortar.

#### 3.2.3. Abrasion Performance

The results of the effect of different filler dosages on the abrasion performancer of PMMA repair mortar maintained at −10 °C for 28 d are shown in Table 5.

As the filler fills the voids of PMMA repair mortar with tiny particle size, it increases the denseness of the mortar, which improves the mechanical performance of the mortar, so the abrasion resistance of PMMA repair mortar is also greatly improved. The abrasive volume and mass of PMMA repair mortar were found to decrease and then increase with the increase of filler dosage. It can be learned from Table 5 that the optimum dosage of the three fillers were 60 phr of fly ash, 40 phr of talc, and 60 phr of wollastonite powder, separately. Compared to the reference group, the abraded volumes were 83 mm^3^, 95 mm^3^ and 107 mm^3^, which were 28.45%, 18.10% and 7.76% lower, and the abraded masses were 0.25 g, 0.27 g and 0.31 g, reduced by 21.88%, 15.63% and 3.13%, respectively. Compactness is the main factor affecting the abrasion performance of PMMA repair mortar, and the PMMA repair mortar under these ratios corresponds to the best mechanical performance, so it has the highest compactness and the best abrasion resistance. The 28 d compressive strength increased at any amount of fly ash, so the abrasion resistance of PMMA repair mortar with fly ash as filler was superior to that without filler.

### 3.3. Anti Salt Freezing Performance

**Figure 6:**
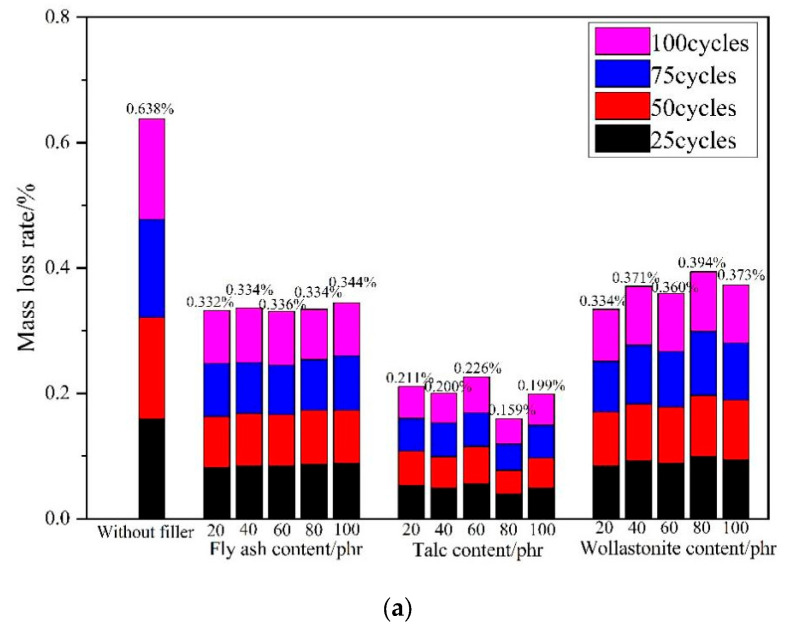

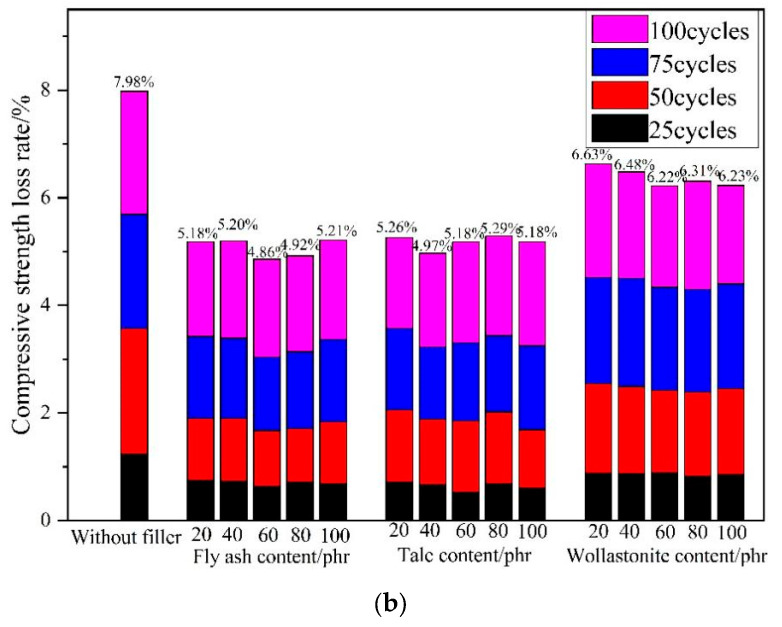
Influence of fillers contents on salt freeze resistance of PMMA repair mortar. (**a**) mass loss rate (**b**) compressive strength loss rate.

In cold regions or in winter, freeze-thaw cycles can lead to microcracks in concrete pavements that have been hardened. Especially when the environment temperature is −10 °C or below, deicing salt is usually sprinkled and spreaded on the pavement. The salt solution causes much more concrete damage than water, and the presence of salt solution on the surface during freezing can cause more serious erosion damage. Since this study is about PMMA repair mortar with a maintenance temperature of −10 °C, it is necessary to investigate the salt freezing performance of PMMA repair mortar. Adopting cyclic conditions of air freezing and 3% NaCl solution thawing to investigate the effect of filler dosage on the rate of mass loss and compressive strength loss of PMMA repair mortar, and the results are shown in Figure 6.

As can be seen in Figure 6, the addition of filler can effectively reduce the rate of mass loss and compressive strength loss to PMMA repair mortar in a number of salt freezing cycles. After 100 cycles of salt freezing, there was a mass loss of 0.638% and a compressive strength loss of 7.98% in the reference group. The lowest mass loss rate of 0.332%, 0.159% and 0.334% were achieved when fly ash was used with a dosage of 20 phr, Talc with a dosage of 80 phr, and wollastonite powder with a dosage of 20 phr, respectively. The loss of compressive strength reached its lowest rate at 4.86%, 4.97% and 6.22% when the dosage of fly ash was 60 phr, talc was 40 phr and wollastonite powder was 60 phr, respectively. Among the three fillers, talc had the best effect on the improvement of salt freezing performance of PMMA repair mortar.

According to the analysis of the mechanism of salt freezing damage [44,45], the overall good resistance of PMMA repair mortar to salt freezing is due to the dense polymer film formed during the molding process that can block the intrusion of foreign media. In addition, the filler’s refinement of the pore size makes the PMMA repair mortar matrix more compact, so it has a stronger blocking capacity and better reduce the expansion stress caused by salt freezing. Talc has an overlapping arrangement of the palace structure, which makes the invasion path of foreign media more tortuous, more difficult to penetrate. Therefore, the PMMA repair mortar with talc as filler has higher anti-salt freezing performance.

### 3.4. Shrinkage

**Figure 7:**
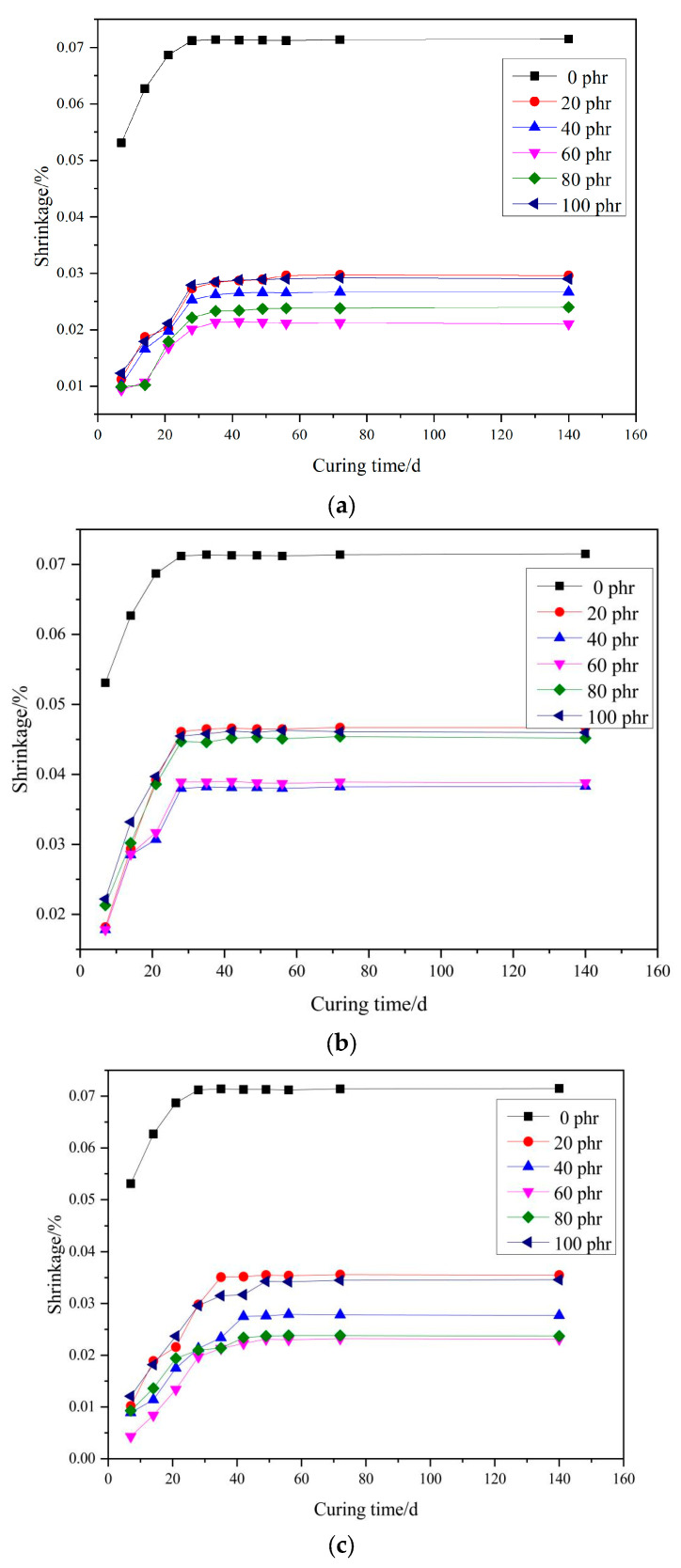
Influence of fillers contents on shrinkage of PMMA repair mortar, (**a**) fly ash (**b**) talc (**c**) wollastonite.

Shrinkage is an important performance indicator of repair mortar, which is related to the volume compatibility of repair mortar and reference mortar after repair. Shrinkage of the newly laid repair mortar will generate internal stresses at the repair interface, and if the shrinkage rate is too large, the repair interface will be prone to cracking and lead to repair failure. Therefore, the effect of filler dosage on the shrinkage of PMMA repair mortar was investigated under the condition of maintenance at −10 °C. The results are shown in Figure 7.

As can be seen from Figure 7, the shrinkage of PMMA repair mortar with added filler is lower than that of PMMA repair mortar without filler, and it can reduce the shrinkage at all ages. The final shrinkage of PMMA repair mortar without filler is about 0.071%. With the increase of filler dosage, the shrinkage of PMMA repair mortar are first reduced and then increased. When the dosage of fly ash is 60 phr, its final shrinkage is about 0.021%, which is 70.42% lower than that of the unfilled mortar. When the dosage of talc is 40 phr, its final shrinkage is about 0.038%, which is 46.48% lower than when no filler is added. When the dosage of wollastonite powder is 60 phr, its final shrinkage is about 0.023%, which is 67.61% lower than when no filler is added.

Among the three kinds of fillers, fly ash has the strongest effect in reducing shrinkage, followed by wollastonite powder and finally talcum powder. The role of filler to reduce shrinkage is, on the one hand, filler particles dispersed in the PMMA resin, the PMMA resin shrinkage played a role in hindering; on the other hand, small particle size filler can be filled in the gap between the PMMA resin and aggregates to improve the denseness of the PMMA repair mortar, the two aspects of the joint effect of the reduction of the shrinkage of the PMMA repair mortar.

### 3.5. Discussion

**Figure 8:**
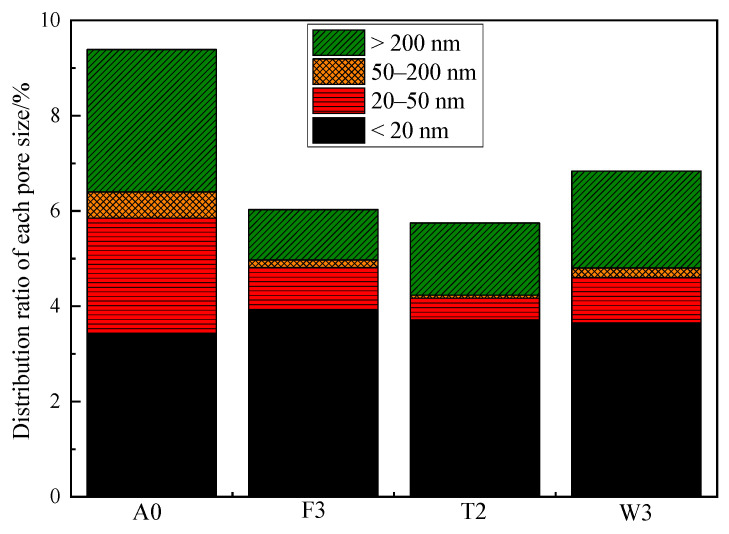
Distribution rate of each pore size of PMMA repair mortar with different ratios.

Mortar is a porous material and pore structure is inseparable and closely related to the macroscopic properties [46,47,48]. For analyzing the influence mechanism of filler on PMMA repair mortar from a microscopic point of view, the porosity of PMMA repair mortar was tested by using piezomercury method. Through the experimental results of the mechanical and durability performance, it was found that for the fillers, 60 phr fly ash, 40 phr talc and 60 phr wollastonite powder had the best comprehensive performance. Therefore, these three mortars and the filler-free PMMA repair mortar were selected for mercury compression testing, and the obtained pore structure parameters and the distribution ratio of each pore size are shown in Table 6 and Figure 8, respectively.

From Table 6, it can be seen that the addition of all three fillers significantly reduced the porosity and average pore size of the mortar. Since the porosity and average pore size of the mortar after mixing the filler are mainly related to the particle size of the filler itself, the effects of the three fillers on the porosity of the PMMA repair mortar are talcu > fly ash > wollastonite powder in descending order, which coincides with the average particle size data of the three fillers in Table 1. However, according to the mechanical property test results, it can be found that although the porosity of PMMA repair mortar mixed with talc is the lowest, but its strength is not the highest. Therefore, although the porosity can reflect the strength, the porosity is not directly proportional to the strength.

Micropores in concrete can be categorized into four types: harmless pores (pore size less than 20 nm), less harmful pores (pore size 20–50 nm), harmful pores (pore size 50–200 nm), and multi-hazardous pores (pore size greater than 200 nm) [49]. Figure 8 shows the histogram of the pore size distribution ratio of each repair mortar, from which it can be seen that the filler can effectively improve the proportion of harmless pores in PMMA repair mortar. It can be observed that the proportion of harmless pores is 65.01%, 64.52% and 53.36% when 60 phr fly ash, 40 phr talc and 60 phr wollastonite powder are incorporated, respectively. Comparing the strength data obtained in 3.2.1, the strength of PMMA repair mortar was the highest when fly ash was used as filler, which corresponded to the largest proportion of harmless pores to the total porosity. Therefore, we can learn that the proportion of harmless pores in the pore structure of mortar is the main factor affecting the strength of mortar.

**Figure 9:**
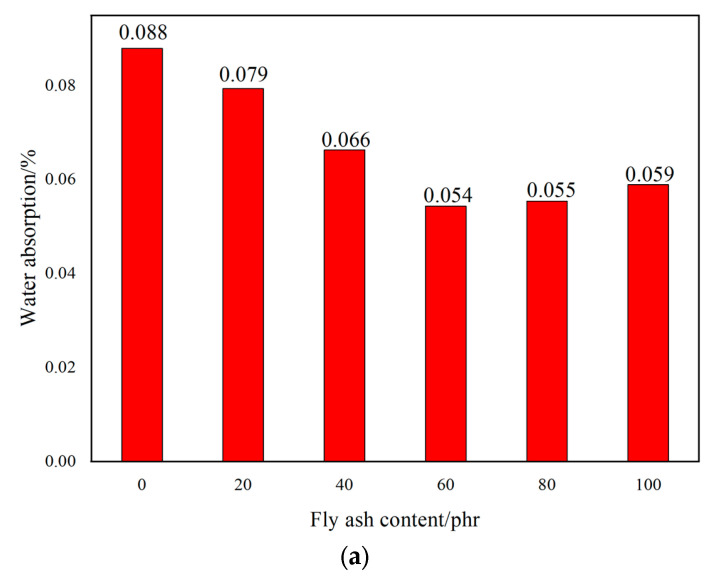

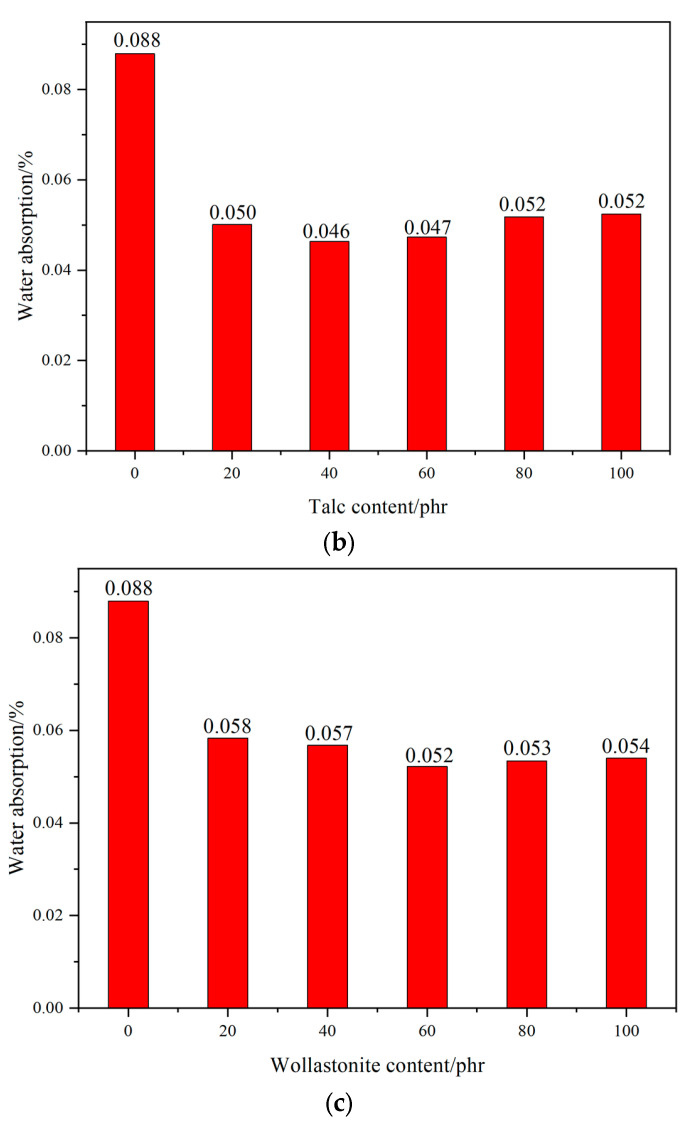
Influence of fillers contents on water absorption of PMMA repair mortar. (**a**) fly ash (**b**) talc (**c**) wollastonite.

Figure 9 represents the effect of filler dosage on the water absorption of PMMA repair mortar after 28 d of curing at −10 °C. It is known that with the increase of filler dosage, the water absorption of PMMA repair mortar all showed a trend of decreasing first and then increasing. Same as the porosity test results, the water absorption of PMMA repair mortar was lowest when the amount of fly ash was 60 phr, talc was 40 phr, and wollastonite powder was 60 phr. The water absorption was reduced by 38.23%, 47.21% and 40.61%, respectively, compared with that when no filler was added. Because of the lamellar structure of the talc, in addition to filling the pore effect, it can also block the pore channel, so the water absorption of PMMA repair mortar with talc is reduced to the greatest extent. Compared to wollastonite powder, fly ash has a smaller average particle size and a higher ability to fill pores. Therefore, the PMMA repair mortar with fly ash has the second highest water absorption, while the PMMA repair mortar with wollastonite powder has the relatively highest water absorption among the three fillers.

**Figure 10:**
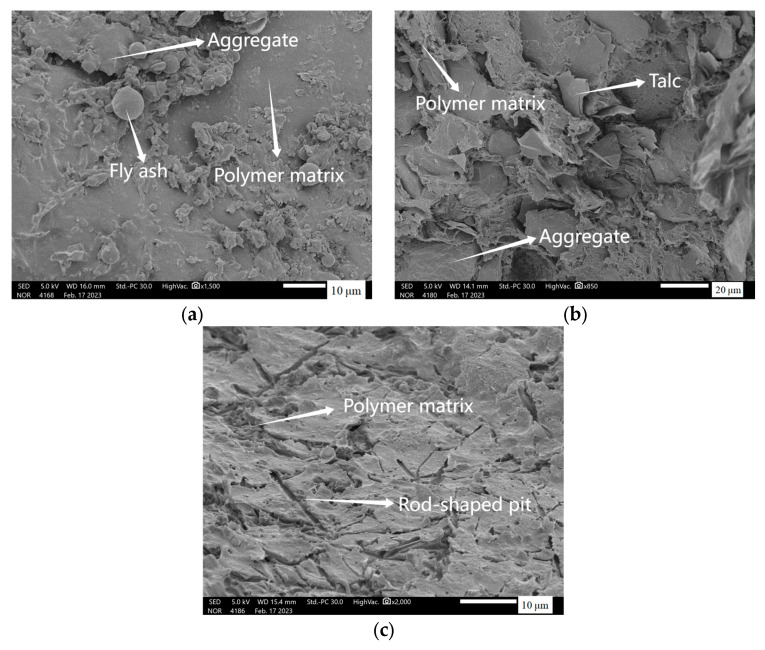
SEM microsgraphs of PMMA repair mortars containing different fillers. (**a**) fly ash (**b**) talc (**c**) wollastonite.

As can be seen from the Figure 10, the fracture surfaces of the three mortars are obviously rough and uneven. Fly ash and talcum powder are embedded in the repair mortar matrix in the form of microspheres and lamellar layers, respectively. And the wollastonite powder in the repair mortar disappears, leaving only its pin-rod-like pits. The filling of filler increases the compactness of PMMA repair mortar. When subjected to external forces, the filler will play an obstructive role in the development of microcracks in the path of the need for additional de-attachment of the filler work and pull-out work to increase the development of microcracks consumed by the energy to impede the propagation of cracks. When subjected to the same torque, fly ash particles and talc particles are still embedded in the mortar, wollastonite powder is “pulled out”, proving that the degree of adhesion between wollastonite powder and PMMA repair mortar is not as high as that of fly ash and talc. From the particle size data of the fillers in 2.1, it can be seen that the average particle size of wollastonite powder is significantly larger than that of fly ash and talc. This is one of the reasons why wollastonite powder has a weaker effect on the strength enhancement of PMMA repair mortar than fly ash and talcum powder.

**Figure 11:**
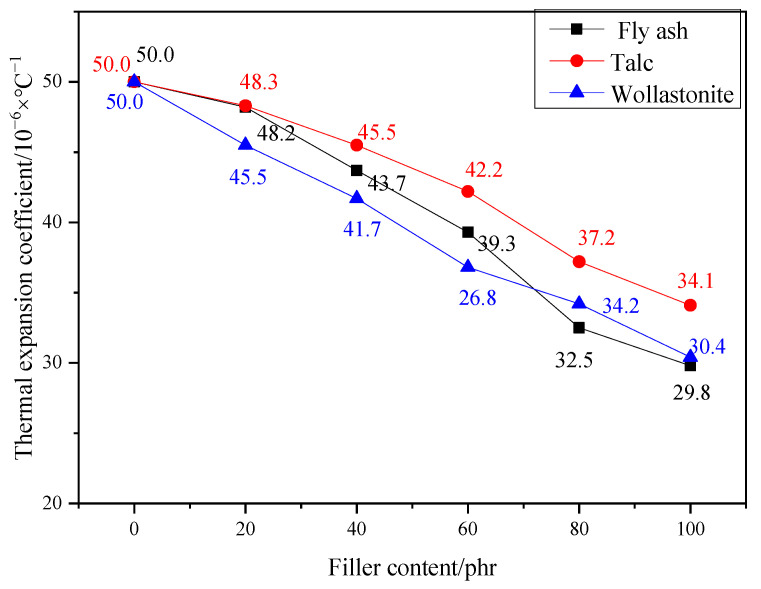
Influence of fillers contents on thermal expansion coefficient of PMMA mortar.

Figure 11 shows the effect of filler amount on the coefficient of thermal expansion of PMMA mortar. The coefficient of thermal expansion of ordinary cement concrete pavement is 8 − 12 × 10^−6^/°C, and the coefficient of thermal expansion of PMMA repair mortar without filler is 50.03 × 10^−6^/°C, which is 4–5 times of the coefficient of thermal expansion of ordinary cement concrete pavement. And when the filler dosage was 100 phr, the coefficients of thermal expansion of mortar doped with fly ash, talc, and wollastonite powder were 29.8 × 10^−6^/°C, 34.1 × 10^−6^/°C and 34.1 × 10^−6^/°C, respectively, which were 40.44%, 31.84%, and 39.24% lower than that of the mortar with no filler added, respectively. When the external temperature changes, if the material in contact with each other can be free deformation, it will not produce internal stress inside the material. But the material is the interaction force and mutual binding force between the two, if the difference in the coefficient of thermal expansion between the two is too large, it will lead to the production of internal stresses in the material, thus affecting the bonding situation between the two [50]. After adding 100 phr filler, the coefficient of thermal expansion of PMMA repair mortar decreased by 30–40% compared with that without filler. The thermal compatibility between PMMA repair mortar and ordinary concrete pavement was significantly improved by adding filler.

Pore structure, water absorption and SEM analysis revealed the role of different types of fillers in repair mortars. On the one hand, fillers filled the internal pores of the mortar, reduced the pore size, decreased the proportion of harmful pores, and provided better densification. On the other hand, the uniformly distributed fillers increased the energy required for the diffusion of microcracks, thus achieving the effect of increasing the strength of the PMMA repair mortar. Finally, the results of the coefficient of thermal expansion test showed that the filler, in addition to improving the performance of the repair mortar itself, can greatly improve the thermal compatibility between the PMMA repair mortar and the cement concrete pavement, and reduce the damage due to temperature stress fatigue.

## 4. Conclusions

In this paper, the effects of fly ash, talc and wollastonite powder on the working performance, mechanical performance and durability of PMMA repair mortar were investigated, and the main conclusions are as follows:

(1) 60 phr of fly ash has the greatest effect on the overall performance of PMMA repair mortar.
(2) All three fillers prolonged the setting time of the PMMA repair mortar. Wollastonite powder had the least negative effect on the setting time of the repair mortar. The larger the dosage of talc and fly ash, the lower the fluidity of the mortar. With the increase in the amount of fly ash, the fluidity of the mortar tended to increase and then decrease.
(3) All fillers improved the mechanical properties of the repair mortar itself to different degrees. Among them, fly ash had the most significant enhancement effect on the mechanical performance. The addition of fillers had no negative effect on the bond strength of the PMMA repair mortar to the reference mortar.
(4) The filler effectively improved the anti-salt-freezing performance of PMMA repair mortar. Thanks to the unique overlapping layer structure of talcum powder, its comprehensive anti-salt freezing performance was the most excellent. Since the filler itself does not shrink, so the shrinkage of the PMMA repair mortar after the addition of the filler was also significantly reduced.
(5) Fillers improve the densification of PMMA repair mortars. The inclusion of fillers greatly improves the thermal compatibility between the PMMA repair mortar and the cement concrete pavement, reducing damage at the bond interface due to temperature stress fatigue.

## Figures and Tables

**Table 1:** Particle size distribution of fillers.

Materials	D10/μm	D50/μm	D90/μm	D (mean)/μm
fly ash	1.06	11.55	47.21	21.44
talc	1.75	8.82	31.82	13.92
wollastonite	2.62	18.58	47.39	22.26

**Table 2:** Raw material ratio of MMA prepolymer/wt%.

MMA	BPO	DOP	PVAc	Styrene
67.38	0.41	20.21	8.40	3.60

**Table 3:** Results of the performance tests of the benchmark PMMA repair mortar.

Fluidity (mm)	Setting Time (min)	Flxural Strength (MPa)			Compressive Strength (MPa)			28 d Bonding Condition	
		1 d	7 d	28 d	1 d	7 d	28 d	Bonding Strength (MPa)	Fracture Surface
220.00	65.00	21.65	23.59	24.79	50.88	60.13	61.79	>6.25	Reference mortar specimen

**Table 4:** Influence of fillers contents on the flexural bonding strength of PMMA repair mortar.

Dosage/phr	Fly Ash		Talc		Wollastonite	
	Flexural Bonding Strength/MPa	Fracture Surface	Flexural Bonding Strength/MPa	Fracture Surface	Flexural Bonding Strength/MPa	Fracture Surface
20	>5.97	M	>6.32	M	>5.99	M
40	>5.88	M	>5.92	M	>5.81	M
60	>6.13	M	>6.04	M	>6.04	M
80	>5.98	M	>6.19	M	>6.17	M
100	>6.01	M	>6.23	M	>5.92	M

Note: “M” in the table represents the fracture on the reference mortar specimen.

**Table 5:** Influence of fillers contents on the wear resistance of PMMA repair mortar.

Sample	Dosage/phr	Chord Length of Abrasion Pit/mm	Abrasion Volume/mm^3^	Abrasion Mass/g
A0	0	24.0	116	0.32
F1	20	22.3	92	0.27
F2	40	22.0	89	0.27
F3	60	21.5	83	0.25
F4	80	21.9	88	0.25
F5	100	23.5	109	0.30
T1	20	23.0	102	0.31
T2	40	22.5	95	0.27
T3	60	22.7	98	0.29
T4	80	23.2	105	0.31
T5	100	23.5	109	0.31
W1	20	23.8	112	0.32
W2	40	23.5	109	0.31
W3	60	23.3	107	0.31
W4	80	24.0	116	0.33
W5	100	24.3	123	0.33

**Table 6:** Pore structure parameters of several PMMA repair mortars.

	Total Pore Volume/mL/g	Average Pore Size/nm	Porosity/%
A0	0.05	18.41	9.39
F3	0.02	10.67	6.03
T2	0.02	10.15	5.75
W3	0.03	12.07	6.84

## Data Availability

All data in this article are listed in this paper. The data presented in this study are available on request from the corresponding author.
